# Complete genome sequence of multidrug-resistant *Photobacterium damselae* subsp. *damselae* OF2, isolated from diseased olive flounder (*Paralichthys olivaceus*)

**DOI:** 10.1128/mra.00456-26

**Published:** 2026-06-30

**Authors:** Hyeong-Ju Kim, Hwa-Gyeong Song, Young-Hyeok Kwon, Tatiana Volkova, Yoseb Jang, Na-Young Kim, Ahran Kim, Timothy J. Bruce, So Young Kang, Sung-Ju Jung, Yoonhang Lee

**Affiliations:** 1 Department of Aqualife Medicine, Chonnam National University 34931, Yeosu, Gwangju, Republic of Korea; 2 South Sea Fisheries Research Institute, National Institute of Fisheries Science, Yeosu, Republic of Korea; 3 Pathology Research Division, National Institute of Fisheries Science, Busan, Republic of Korea; 4 School of Fisheries, Aquaculture, and Aquatic Sciences, Auburn University 1383, Auburn, Alabama, USA; Fluxus Inc., Sunnyvale, California, USA

**Keywords:** *Photobacterium damselae *subsp. *damselae*, multiple antibiotic resistance, olive flounder, complete genome, resistance plasmid

## Abstract

Here, we report the complete genome sequence of a multidrug-resistant *Photobacterium damselae* subsp. *damselae* strain OF2, isolated from diseased olive flounder (*Paralichthys olivaceus*) in South Korea. Oxford Nanopore sequencing yielded two circularized chromosomes and a plasmid carrying antimicrobial resistance genes.

## ANNOUNCEMENT

*Photobacterium damselae* subsp. *damselae* (*Pdd*) is a causative agent of photobacteriosis, an important bacterial disease affecting marine aquaculture worldwide, including olive flounder (*Paralichthys olivaceus*) farming in South Korea (1, 2). In Korean aquaculture, various antibiotics are widely used for disease control; however, the emergence of multidrug-resistant strains poses a significant challenge to effective treatment (3). Strain OF2 was isolated from a diseased olive flounder farm in Wando (34.31° N, 126.76° E), South Korea, in 2022. Kidney was aseptically collected from the fish, streaked onto tryptic soy agar (Difco, USA) supplemented with 1% NaCl, and incubated aerobically at 28°C for 24 h. The isolate was identified as *Pdd* by multiplex PCR assay targeting the *ureC* and 16S rRNA genes, as previously described by Osorio et al. (4). Disk diffusion testing was performed based on Clinical and Laboratory Standards Institute guideline VET03 (5). The isolate exhibited no inhibition zones against ampicillin, amoxicillin, oxytetracycline, oxolinic acid, and enrofloxacin, indicating a multidrug-resistant phenotype.

For sequencing, the bacterium was inoculated into tryptic soy broth (Difco) supplemented with 1% NaCl and incubated at 28°C with shaking at 180 rpm. Bacterial cells were harvested at 12 h, and gDNA was extracted using the Wizard HMW DNA Extraction Kit (Promega, USA). DNA concentration was measured using a Qubit 4 fluorometer (Thermo Fisher Scientific, USA) with the Qubit 1X dsDNA High Sensitivity Assay Kit (Thermo Fisher Scientific). Sequencing libraries were prepared using Rapid Barcoding Kit 24 v14 (Oxford Nanopore Technologies [ONT], UK). High-molecular-weight DNA was used without shearing or size selection. Sequencing was performed on an R10.4 flow cell (ONT) using a MinION Mk1D device (ONT). Basecalling was performed using Dorado v1.4.0 (ONT) with super-accuracy mode. Subsequently, raw reads were filtered using NanoFilt v2.8.0 (6) (*Q* ≥ 10 and read length ≥2,500 bp), assembled *de novo* using Flye v2.9.5 (7), which also confirmed the circularity of the replicons, and polished with Medaka v2.2.0 (ONT). The contigs were reoriented to begin at the *dnaA* gene using dnaapler v1.3.0 (8). Genome completeness was assessed using CheckM v1.2.5 (9). Average nucleotide identity (ANI) was calculated using FastANI v1.34 (10). The genome was annotated using the NCBI Prokaryotic Genome Annotation Pipeline v6.10 (11), and antimicrobial resistance genes (ARGs) were identified using AMRFinderPlus v4.2.7 (12). All analyses were performed using default parameters unless otherwise noted.

Sequencing yielded 70,371 reads totaling 455,286,085 bp, with a read N50 of 4,346 bp and a median *Q* score of 23.3. The complete genome comprised two circular chromosomes (3,263,786 and 1,427,031 bp) and one circular plasmid (212,570 bp), with an average coverage of 93× (Table 1). ANI analysis showed that strain OF2 shares 98.60% identity with *Pdd*-type strain CIP 102761 (GenBank accession numbers ADBS01000001.1–ADBS01000008.1). BLASTn analysis of the plasmid showed 99.8% nucleotide identity (70% query coverage) with a plasmid from *Pdd* strain PDD0906 (GenBank accession number CP077688.1). A total of seven ARGs were identified, including *qnrS2* and *bla_PSE-4_*, present on both the chromosome and plasmid, and *tet(B)* and *tet(M)*, which were exclusively plasmid-borne. These findings are consistent with the multidrug-resistant phenotype of strain OF2.

**TABLE 1:** Genome features and antimicrobial resistance genes of *Photobacterium damselae* subsp. *damselae* strain OF2

	Length (bp)	Coverage	GC (%)	Number of genes				Antimicrobial resistance genes (locus_tag)
				CDS	rRNA	tRNA	ncRNA	
Chromosome 1	3,263,786	93×	41.6	2,737	62	200	4	*qnrS2* (AC1KR8_10430)
Chromosome 2	1,427,031	69×	39.0	1,184	0	11	0	*bla_PSE-4_* (AC1KR8_16355)
Plasmid	212,570	285×	39.0	188	0	0	0	*bla_PSE-4_* (AC1KR8_21120) *tet(B)* (AC1KR8_21870) *tet(M)* (AC1KR8_21895) *qnrS2* (AC1KR8_21435 and AC1KR8_21750)
Total	4,903,387	93×	40.7	4,109	62	211	4	–^*a*^

^*a*^ –, not applicable.

## Data Availability

The complete genome sequences of this project have been deposited in the NCBI GenBank (accession numbers JBWWNS000000001.1 [chromosome I], JBWWNS000000002.1 [chromosome II], and JBWWNS000000003.1 [plasmid], within BioProject accession number PRJNA1449044 and BioSample accession number SAMN57108698). The raw Oxford Nanopore read data can be found under accession number SRR37937613.
